# Managing Localization Uncertainty to Handle Semantic Lane Information from Geo-Referenced Maps in Evidential Occupancy Grids †

**DOI:** 10.3390/s20020352

**Published:** 2020-01-08

**Authors:** Chunlei Yu, Veronique Cherfaoui, Philippe Bonnifait, Dian-ge Yang

**Affiliations:** 1 State Key Laboratory of Automotive Safety and Energy, School of Vehicle and Mobility, Tsinghua University, 10084 Beijing, China; yuchunlei_tsinghua@163.com; 2 Sorbonne Universités, Université de Technologie de Compiègne, CNRS Heudiasyc UMR 7253, 60203 Compiegne, France; veronique.cherfaoui@hds.utc.fr (V.C.); philippe.bonnifait@hds.utc.fr (P.B.)

**Keywords:** evidential occupancy grid, uncertainty, lane grid, prior map, semantic

## Abstract

Occupancy grid is a popular environment model that is widely applied for autonomous navigation of mobile robots. This model encodes obstacle information into the grid cells as a reference of the space state. However, when navigating on roads, the planning module of an autonomous vehicle needs to have semantic understanding of the scene, especially concerning the accessibility of the driving space. This paper presents a grid-based evidential approach for modeling semantic road space by taking advantage of a prior map that contains lane-level information. Road rules are encoded in the grid for semantic understanding. Our approach focuses on dealing with the localization uncertainty, which is a key issue, while parsing information from the prior map. Readings from an exteroceptive sensor are as well integrated in the grid to provide real-time obstacle information. All the information is managed in an evidential framework based on Dempster–Shafer theory. Real road results are reported with qualitative evaluation and quantitative analysis of the constructed grids to show the performance and the behavior of the method for real-time application.

## 1. Introduction

Grid-based environment modeling has become a popular perception paradigm since its first introduction in [1]. Compared with object-based approaches such as [2,3], the grid-based method has several advantages since it is straightforward to integrate heterogeneous sensor information and it relaxes data-association problems which can be especially hard to handle in complex and dynamic environments. There exists a large literature regarding to grid-based environment modeling for robotics applications and for autonomous vehicles. In [4], the authors have presented an occupancy-elevation grid mapping technique for autonomous navigation application. In [5], the occupany grid is constructed to identify different shape of objects by applying sensor fusion. In [6], the authors have presented a universal grid map library for grid construction. In [7], the authors introduced an advanced occupancy grid approach to enable the robust separation of moving and stationary objects. In [8], a generic architecture for perception in dynamic outdoor environment by applying grid-based SLAM (Simultaneous Localization and Mapping) is presented. In [9], a grid-based approach is proposed for ADAS (Advanced Driving Assistance System) application. In [10], a real-time algorithm for grid-based SLAM and detection of objects is presented.

Some works tried to integrate dynamic information into grids to get a more complete representation of the environment. In [11], the space occupied by static and dynamic objects is distinguished by adopting a sequential fusion formalism. In [12], the authors proposed a complete semantical occupancy grid mapping framework involving a new interpolation method to incorporate sensor readings in a Bayesian way. In [13], a Bayesian filter approach was adopted to estimate the state of the occupancy grid with velocity information.

This paper deals with perception grids for the navigation of road vehicles. The road space is classically classified as occupied (by obstacles) or free. However, for autonomous navigation on public roads, this road space description is not sufficient as the host vehicles should be able to know which space is free to go with authorization. For this purpose, lane-level information should be provided. A vehicle typically has two options when facing a static obstacle in its own lane: (i) lane-keeping and stop or (ii) lane changing. For the second option, the host vehicle needs semantic road rules deduced from lane marking information to evaluate if the space is accessible or not. Thus, the road must be modeled semantically to represent the accessibility of the space.

Many lane detection methods have been proposed and developed using image processing. In [14], a robust and real-time approach to detect lane markers was proposed. In [15], the authors proposed a robust multilane detection and tracking method using LiDAR and mono-vision data. In [16], based on spatiotemporal images, the authors proposed a method for lane detection, which proved to be more robust and efficient. In [17], an implementation of semantic image segmentation to enhance LiDAR-based road and lane detection was presented. In [18], using a proposed region of interest, the authors managed to reduce the calculation and high noise level for lane detection. Images are typically segmented into road, obstacles, sky, etc. These methods can be strongly influenced by weather conditions and by the quality of lane markings. Deep learning-based lane detection methods have shown superior performance over traditional methods. In [19], the authors proposed a Dual-View Convolutional Neural Network framework. Reference [20] extended the framework of deep neural network by accounting for the structural cues. In [21], a unified end-to-end trainable multi-task network that jointly handles lane and road marking detection and recognition was proposed. The approach is guided by a vanishing point under adverse weather conditions. Reference [22] proposed a sequential end-to-end transfer learning method to estimate left and right ego lanes directly and separately without any postprocessing. However, they usually perform well only when the road conditions are similar to those used in the training datasets.

We propose to tackle this problem by using geo-referenced maps. Prior map information has already been used for road object extraction, since it provides cues about the existence and location of on-road objects [23,24,25]. In [24], the authors used an open-source dataset for vehicle detection tasks with geographic information. In [23], the authors have taken advantage of the maps for both localization and perception. With a map-aided pose estimation, they proposed an obstacle detection method based on the comparison between the image acquired by an on-board camera and the image extracted from a 3D model. Now we can have access to very detailed and accurate geo-referenced databases, which provide rich information for autonomous navigation. Using these maps, lane-level attributes describing the structure of the road is available. For this purpose, a localization system is mandatory. In [26,27], a prior map was considered to be a virtual sensor, providing information about the space occupied by infrastructures, buildings, etc. With an accurate pose estimation, the authors converted the extracted information into a local perception map. This perception map was then fused with a local occupancy grid generated from the on-board sensor data. In [28], a holistic 3D scene understanding method was proposed based on geo-tagged images which allows joint reasoning about 3D object detection, pose estimation, semantic segmentation as well as depth reconstruction from a single image. Large-scale crowd-sourced maps were used to generate dense geographic, geometric and semantic priors. In [29], an algorithm for road detection was proposed using Geographical Information Systems (GISs). The priors are obtained by building a road map using information such as the type of the road and the number of lanes, retrieved from a database and then by projecting the road map onto the vehicle frame.

Although geo-referenced maps provide valuable prior semantic information, a real difficulty remains in tackling the uncertainties coming from estimation errors on the pose of the vehicle and the errors on the map features arising from the mapping process. In this paper, we suppose that the prior map is very accurate in comparison with the localization errors thanks to the use of high-grade devices used in the data acquisition process and refined post-treatments. Localization is therefore the main source of uncertainty in this process.

Based on this discussion, this work has the following contributions. We first propose a method to integrate semantic lane information from a prior map into grid–based environment models by taking into account explicitly the localization error of the vehicle. In previous work, we have presented some related results concerning the construction of grids based on prior maps[30]. In this paper, we present extensive theoretical and experimental results to show further the interests of our approach. Secondly, an approach based on Dempster–Shafer theory is presented to fuse this semantic map with an occupancy map built from on-board sensing information.

The paper is organized as follows. In Section 2, the approach to construct semantic lane grid based on prior map and uncertain pose is introduced. In Section 3, the fusion of occupancy grid and semantic lane grid based on the Dempster–Shafer theory is presented. In Section 4, real road results are reported and analyzed. Finally, in Section 5, we draw conclusions.

## 2. Semantic Grids Using Maps and Uncertain Localization

### 2.1. Semantic Grid with Lane Information

In occupancy grids, each cell contains probabilities quantifying the belief that there exists or not an obstacle in the cell. So, the possible states are Free or Occupied. Inspired by occupancy grids, we propose to define spatial grids in which one can interpret the lane information as cell values which have semantic meanings needed by the trajectory planner of the navigation system. The simplest cell states which permit the host vehicle to perform lane-level navigation can be defined as $\left\{Ego,\;Accessible,\;Forbidden\right\}$ in which Ego is the current lane and the two other states define the traffic rules. Differencing between the current lane and the other ones of the carriageway with the same driving direction, is important for lane-keeping or overtaking applications. The Forbidden state defines not only the lanes which are not permitted but also the regions outside of the road.

**Figure 1:**
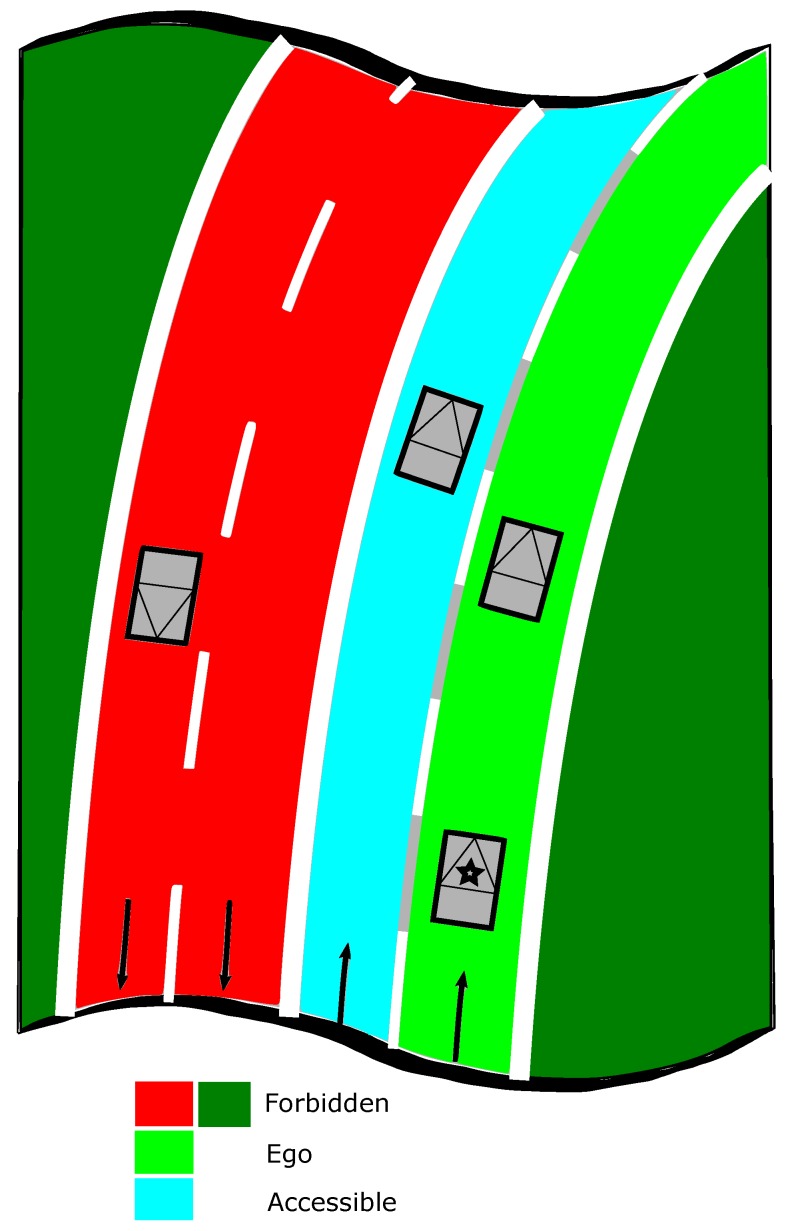
The lane state depends on the host vehicle position depicted with a star. Ego lane in bright green, Accessible lane in cyan, the Forbidden areas in red and dark green.

Figure 1 illustrates the semantic road rules information. The host vehicle is marked with a star on it, the Ego lane is depicted in bright green, the Accessible lane in cyan, the Forbidden areas (which include both the forbidden lanes and regions outside of the current road and the forbidden lanes on opposite direction) are painted in red and the outside road regions on dark green. The boundaries between these regions are the lane markings that implies the traffic rules. As shown in Figure 1, the state of lanes and thus the state of grid cells depends on the position of the host vehicle.

**Figure 2:**
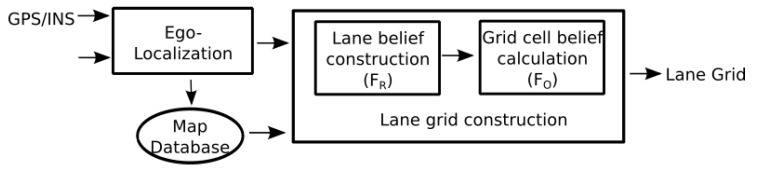
Lane Grid construction process.

To obtain the road and the lane boundary information, we use an accurate map constructed beforehand. Figure 2 illustrates the construction process of the proposed semantic lane grid. The Ego-localization step outputs the 2D pose estimate (with uncertainty) of the host vehicle. One should note that two sources of uncertainty intervene in the construction process, which come respectively from the pose estimation step and from the map. In our approach, we make the hypothesis that the map is accurate (or with an error that is negligible compared to the pose estimate) and with no attribute error thanks to the high-grade sensors used in the map construction process. The pose uncertainty is the predominant uncertainty which must be taken into account. For that, we are interested in studying an evidential approach which is proposed based on Dempster–Shafer theory. The lane grid construction process contains two steps as shown in Figure 2, which are respectively lane belief construction and grid cell belief construction. These two steps will be explained in Section 2.2 and Section 2.3.

**Figure 3:**
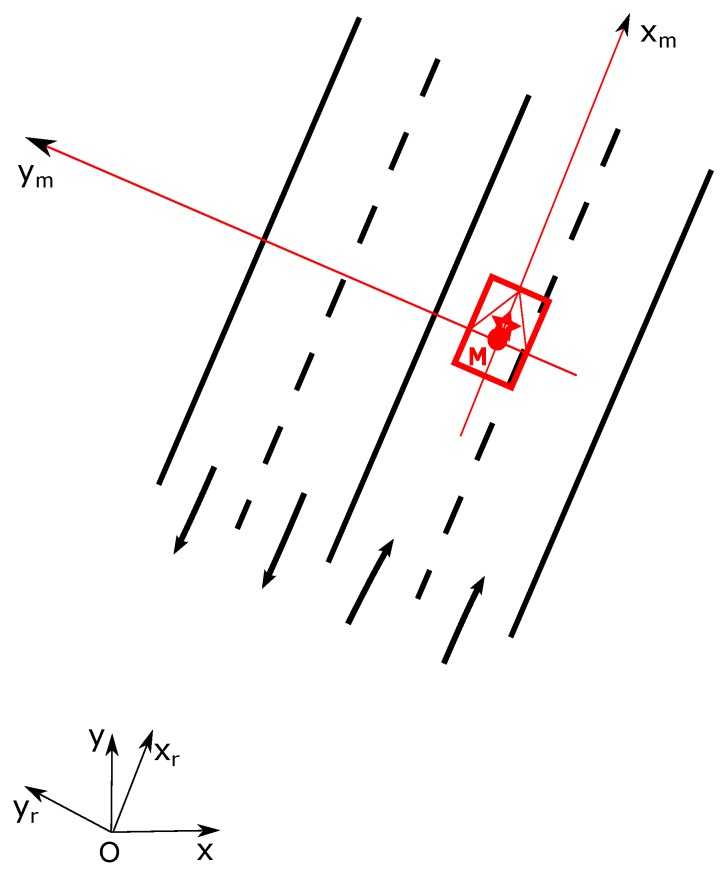
Frames definition.

To facilitate the illustration of our approach, let us define different coordinate systems. Let denote $F_{O}$ as the global frame in which the prior map is defined, with an origin *O*, *x* and *y* pointing to East and North. We also use a road-oriented frame $F_{R}$ [31] which has the same origin *O* of the global frame but with the x-axis pointing in the direction of the road. The working frame of vehicle $F_{M}$ is defined at the center of the vehicle’s rear axle with an origin called *M*. One needs to note that *M* is not a deterministic position in $F_{O}$ or $F_{R}$ because of the estimation uncertainty. Figure 3 shows the coordinate frames definition.

### 2.2. Lane Belief Distribution Construction

The *lane belief distribution* is a characterization of the status of the lanes based on the estimated pose of the host vehicle and its uncertainty.

#### 2.2.1. Uncertainty in the Construction of Lane Belief Distribution in a Road-Oriented Frame

As shown in Figure 2, the first stage for building a lane grid consists of the construction of the lane belief distribution which is here carried out in the road-oriented frame $F_{R}$.

**Figure 4:**
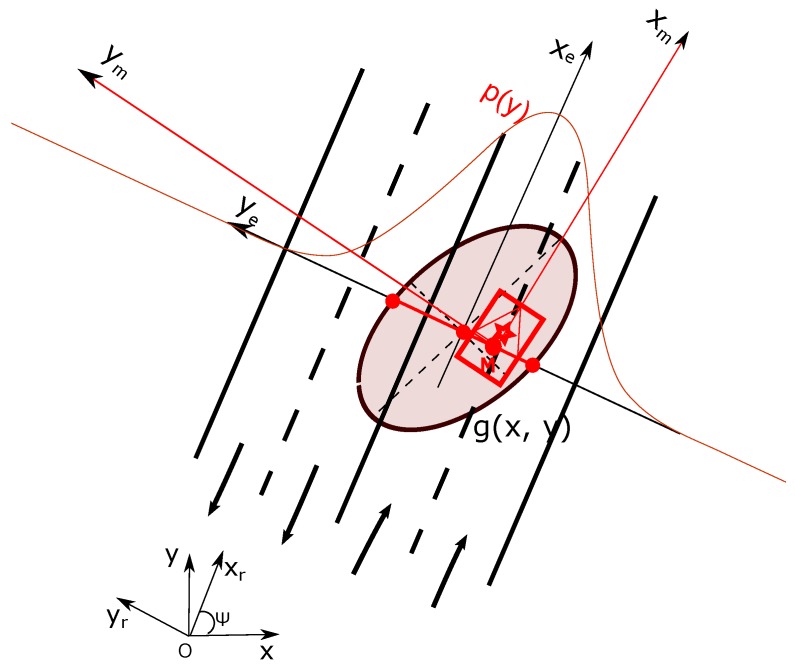
Uncertainty of the estimated pose.

The lane states are characterized according to semantic road rules and depends on which lane the host vehicle is located. This requires the lateral position knowledge of the vehicle relatively to the road. The localization system estimates the position of the vehicle in $F_{O}$ and its uncertainty can be represented by a 2D ellipse as shown in Figure 4. If we consider the local estimated road-oriented frame $\left(x_{e,\;}y_{e}\right)$ shown in Figure 4, the lateral uncertainty of the pose estimation for the construction of the lane belief distribution is perpendicular to the lane or road direction, i.e., along the axis $y_{e}$. This uncertainty characterizes laterally the position of the vehicle relatively to the lanes. The longitudinal uncertainty in the road-oriented frame is not related to the process of determining on which lane the vehicle is localized. The lateral uncertainty is represented by p(y) in Figure 4.

Suppose that the estimation uncertainty is represented by a covariance matrix

$${}^{O}P=\left[\begin{array}{cc} {}^{O}p_{11} & {}^{O}p_{12}\\ {}^{O}p_{12} & {}^{O}p_{22} \end{array}\right]$$

which is defined in $F_{O}$ and is obtained from the ego-localization process. The transformation of this uncertainty into the road-oriented frame $F_{R}$ is given by:

(1) $${}^{R}P=\left[\begin{array}{cc} {}^{R}p_{11} & {}^{R}p_{12}\\ {}^{R}p_{12} & {}^{R}p_{22} \end{array}\right]=R\cdot {}^{O}P\cdot R^{T}$$

where $R=\left[\begin{array}{cc} cos(\psi ) & sin(\psi )\\ -sin(\psi ) & cos(\psi ) \end{array}\right]$ is the rotation matrix, ψ is the heading of the road.

Then the lateral Gaussian distribution’s standard deviation in $F_{R}$ is given by (see [32] for details):

$$\sigma_{R}=\sqrt{{}^{R}p_{22}}\cdot \left(1-{\left(\frac{{}^{R}p_{21}}{\sqrt{{}^{R}p_{11}}\cdot \sqrt{{}^{R}p_{22}}}\right)}^{2}\right)$$

#### 2.2.2. Multi-Hypothesis Approach

The objective now is to quantify the belief supporting the lane distribution over the road knowing the pose uncertainty and the configuration of lanes in the map.

Let denote:

- nl the number of lanes (extracted from the map)
- Ω a frame of discernment of the space with Ω={Ego,Accessible,Forbidden}.
- B(i,A) the belief of state A,A∈Ω for lane *i*.
- $H_{i}$ the hypothesis that lane *i* is the Ego lane
- nh the number of hypotheses $H_{i}$

The model takes into account all the possibilities concerning which lane is Ego, and based on each possibility a hypothesis is proposed and the belief is calculated as the integral of the pose distribution over the lane. For instance, the belief for the hypothesis $H_{i}$ that lane *i* is Ego lane is:

$$B_{H_{i}}\left(i,\;Ego\right)=P\left(Lane\;i=Ego\right)=\int_{RightMarking_{i}}^{LeftMarking_{i}}p\left(y\right)dy$$

with $i\in \left[1,nl\right]$.

With the hypothesis that the map is accurate and with no error of attribute, this belief is propagated to the other lanes of the road. The state *A* ( Accessible or Forbidden) is deduced from the lane markings:

$$B_{H_{i}}\left(j,\;A\right)=B_{H_{i}}\left(i,\;Ego\right)\;\;\forall j\neq i,\;A\in \Omega ,\;A\neq Ego$$

**Figure 5:**
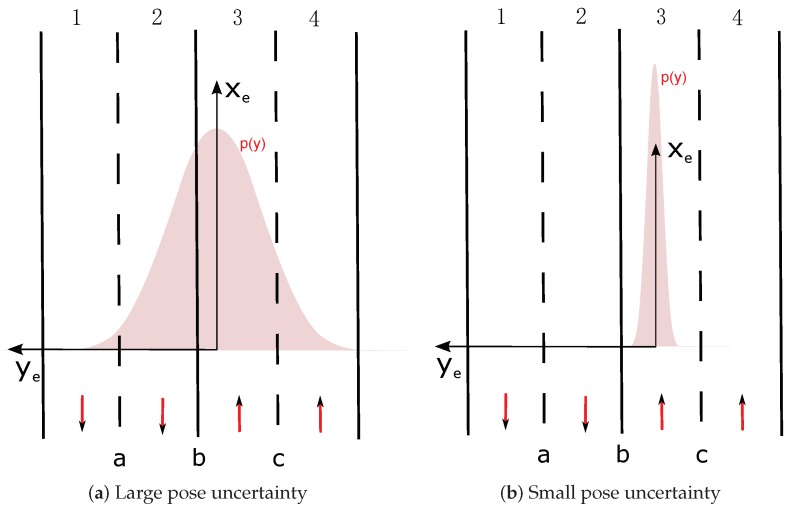
Examples of pose uncertainty and lane belief distribution.

The algorithm considers every lane in which the host vehicle can be located. If the number of possibilities is nh, the belief supporting that particular hypothesis $B_{H_{k}}$ , $k\in \left[1,nh\right]$ is calculated. For example, in Figure 5a nl=4 and nh=4 and in Figure 5b nl=4 and nh=1.

**Figure 6:**
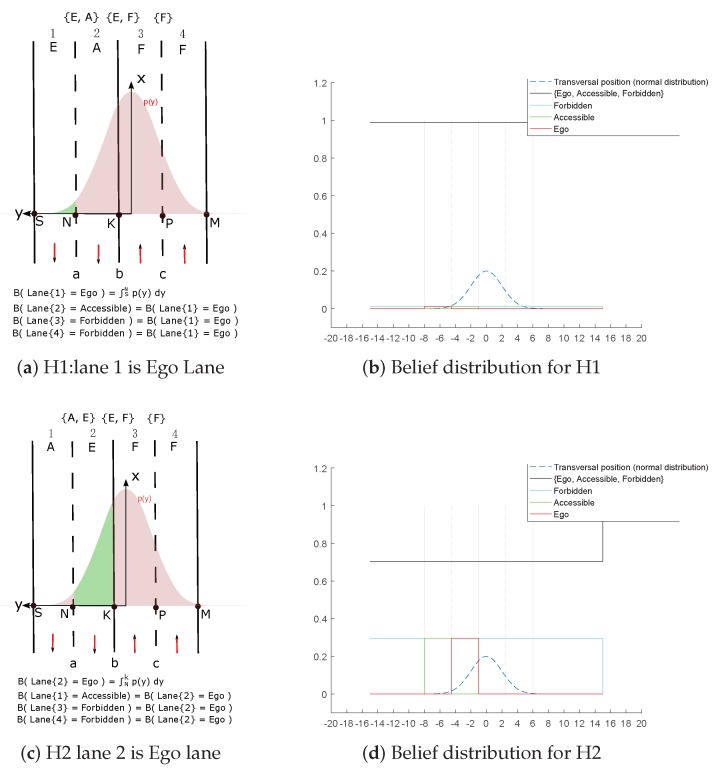

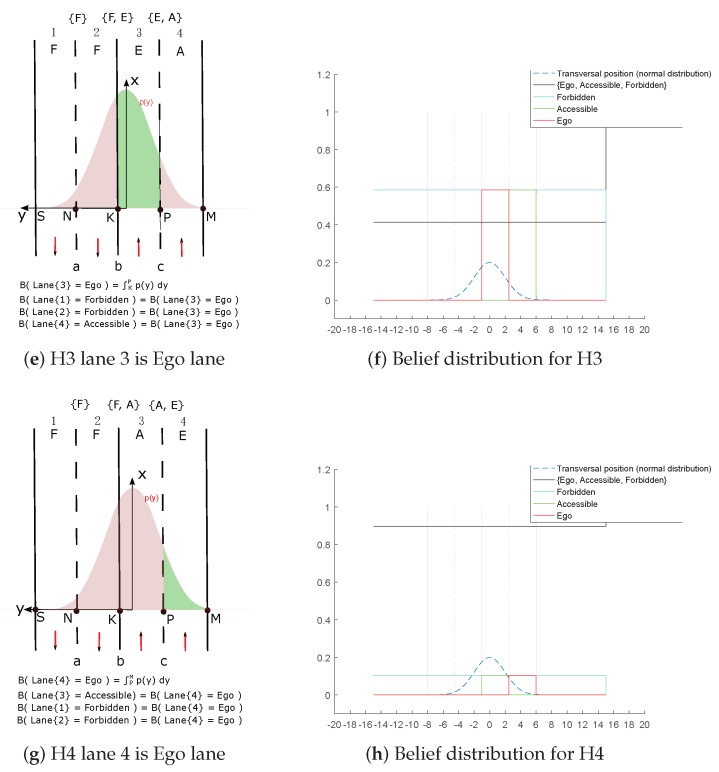
Multi-hypothesis algorithm illustration.

We illustrate our model based on the situation shown in Figure 5. The position has a large lateral uncertainty and the Gaussian distribution covers multi-lanes. That leads exactly to four hypotheses: “H1: lane 1 is Ego lane”, “H2: lane 2 is Ego lane”, “H3: lane 3 is Ego lane”, “H4: lane 4 is Ego lane”. Every hypothesis is shown in Figure 6. Ego lane’s belief is drawn in green color inside the pose distribution. *A* means Accessible, *E* means Ego, *F* means Forbidden.

There exists the rare case that the true pose of the host vehicle lies outside the road. Our method does not ignore this possibility. The two regions on the left and right side of the road are considered to be two independent spaces in our implementation. This treatment can also guarantee the unity of the calculated belief.

The algorithm considers every case in which the host vehicle is located in each lane, then computes for each case the belief supporting that particular hypothesis.

#### 2.2.3. Belief Accumulation

To compute the final lane belief distribution, we accumulate the respective belief of the hypotheses to the correspondent proposition. This accumulation corresponds to the law of total probability and we can apply this accumulation strategy thanks to the fact that all hypotheses come from the same information source: the pose distribution.

$$B\left(j,\;A\right)=\sum_{k=1}^{nh}B_{H_{k}}\left(j,\;A\right)\;\forall j\in \left[1,nl\right],\;\forall A\in \Omega$$

**Figure 7:**
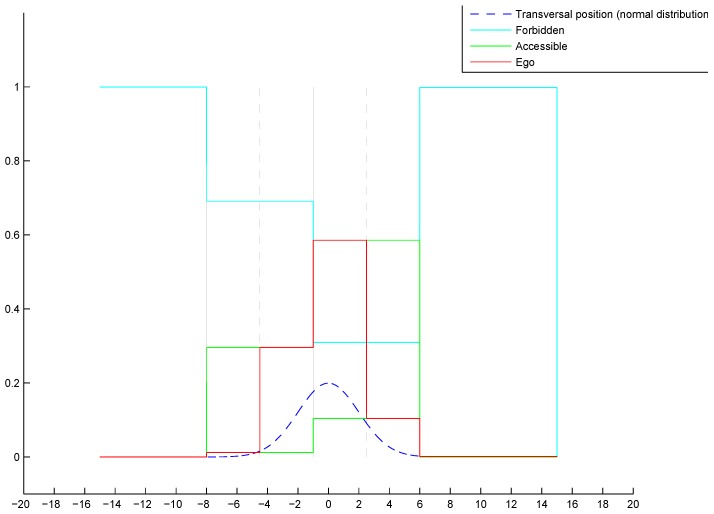
Final lane belief distribution.

The final belief distribution of the previous example is shown in Figure 7. The color code is shown in the legend in the figure. At first sight, one can remark that the proposed multi-hypothesis method leads to Ego belief for every lane because of the pose distribution, all the other belief levels are deduced from this base. The belief accumulation is mostly highlighted from the Forbidden mass outside the road. The pose distribution in this case is limited to the road area, and we can remark that all the belief goes to the Forbidden state outside this area, in line with reality.

### 2.3. Grid Cell Belief Calculation

The objective is to compute now the distribution of belief for each cell of the grid. Both probabilistic and evidential approaches are illustrated to tackle the grid cell uncertainties. In this section, we show first the propagation of the pose uncertainty over the grid. The two methods to deal with uncertainty will be both illustrated in the following sections. The term Belief adopted here in the schema of Figure 2 can have different meanings depending on the considered approach. In the probabilistic one, Belief refers to Probability. In the evidential framework, Belief means Mass, since Mass is the basic belief assignment in the belief function theory.

#### 2.3.1. Uncertain Location of the Grid Cells

**Figure 8:**
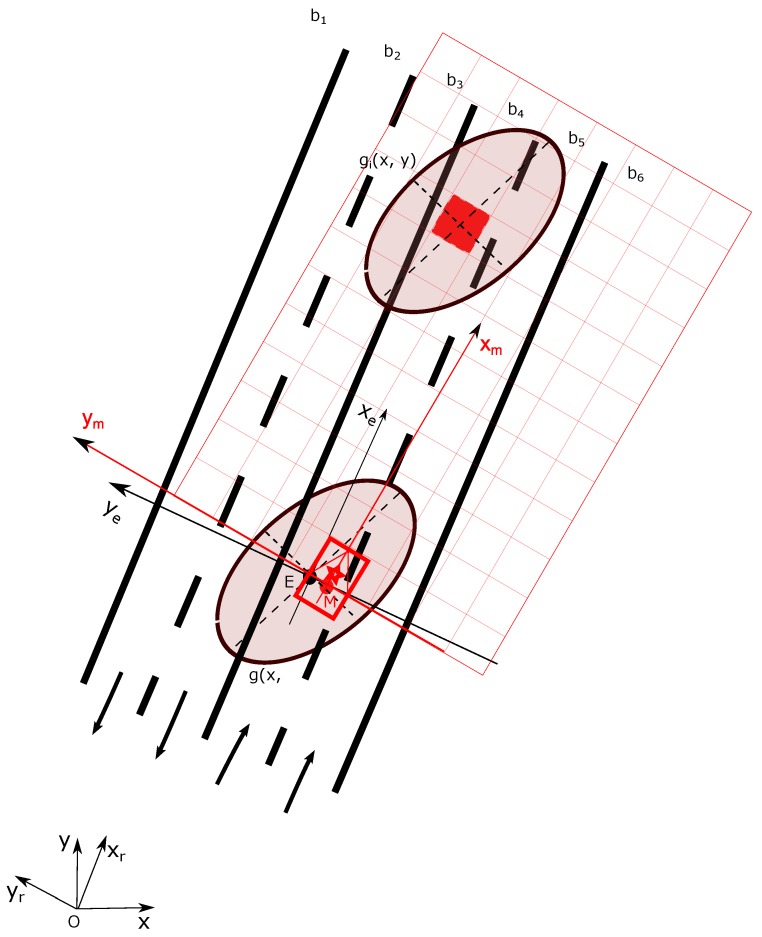
Lane cell mass computation.

Figure 8 gives an illustrative example. $b_{1}∼b_{6}$ represent the lane belief distribution. Let imagine that the true position of the vehicle is located at *M* and the lane grid is shown in red. One can also remark that in a 2D situation, another important source of uncertainty for lane grid cells is the heading angle of vehicle due to some unavoidable estimation error.

For a cell *i* (red cell in Figure 8) let denote its uncertainty $g_{i}\left(x,\;y\right)$ which depends on its position in the local vehicle coordinate $F_{M}$. For simplification from now on, we consider one cell as a point. The discussion can be easily extended to the cell’s four corner points without loss of generalization.

The coordinates of the cell *i* in $F_{M}$ are

$${}^{M}X_{i}=\left[\begin{array}{c} {}^{M}x_{i}\\ {}^{M}y_{i} \end{array}\right]$$

Transformed into the global coordinate $F_{O}$, the coordinates are:

(2) $${}^{O}X_{i}=\left[\begin{array}{c} {}^{O}x_{i}\\ {}^{O}y_{i} \end{array}\right]{=}^{O}R_{M}\;\cdot \;\left[\begin{array}{c} {}^{M}x_{i}\\ {}^{M}y_{i} \end{array}\right]+\left[\begin{array}{c} {}^{O}x_{M}\\ {}^{O}y_{M} \end{array}\right]$$

where ${}^{O}R_{M}$ represents the rotation matrix from $F_{M}$ to $F_{O}$,

$${}^{O}R_{M}=\left[\begin{array}{cc} \cos(\theta ) & -\sin(\theta )\\ \sin(\theta ) & \cos(\theta ) \end{array}\right]$$

$\left[\begin{array}{c} {}^{O}x_{M}\\ {}^{O}y_{M} \end{array}\right]$ is the position of *M* in global frame. This position has the uncertainty represented by the ellipse g(x,y). One should note that θ is the heading angle which also involves some estimation uncertainty.

To analyze the uncertainty of the cell in the global frame, one can see from Equation (2) that the position depends on five variables: ${(}^{O}x_{M},{\;}^{O}y_{M},\;\theta )$ is the 2D estimated pose of the vehicle in global frame, ${(}^{M}x_{i},{\;}^{M}y_{i})$ is the position of the cell in the local vehicle frame $F_{M}$. ${(}^{M}x_{i},{\;}^{M}y_{i})$ have no uncertainty since the position of grid cells are precisely known. Hence, one can conclude that in the global frame, the position uncertainty of one cell results from the estimated 2D pose of the host vehicle ${(}^{O}x_{M},{\;}^{O}y_{M},\;\theta )$.

To understand the effect of the uncertainties transferred from the pose, we suppose for simplification that the heading angle θ is decorrelated from the position ${(}^{O}x_{M},{\;}^{O}y_{M})$ for illustration purpose.

First, let suppose that the heading angle (θ) has no uncertainty, one can deduce that the position of the cell ${}^{O}X_{i}$ has linear relation with respect to the vehicle position, thus

$$Var{(}^{O}X_{i}|var\left(\theta \right)=0)=Var\left(\left[\begin{array}{c} {}^{O}x_{M}\\ {}^{O}y_{M} \end{array}\right]\right),$$

which shows that the covariance matrix of cell position is identical with the covariance of the vehicle’s position. Thus, this source of uncertainty propagates uniformly to all the cells in the grid.

On the other hand, if we take into account an uncertain heading angle while the position of the vehicle is precisely known, then we can develop Equation (2) into

(3) $${}^{O}X_{i}\;={}^{O}R_{M}\;\cdot \;\left[\begin{array}{c} {}^{M}x_{i}\\ {}^{M}y_{i} \end{array}\right]+\left[\begin{array}{c} {}^{O}x_{M}\\ {}^{O}y_{M} \end{array}\right]=h\left(\theta \right)$$

then the covariance of ${}^{O}X_{i}$ can be computed as

(4) $$\begin{array}{ccc} Var{(}^{O}X_{i}|Var\left(\left[\begin{array}{c} {}^{O}x_{M}\\ {}^{O}y_{M} \end{array}\right]\right) & = & 0)\;=\left[\frac{dh}{d\theta }\right]\cdot \;var\left(\theta \right)\;\cdot \;{\left[\frac{dh}{d\theta }\right]}^{T}\\ & & =var\left(\theta \right)\cdot \left[\begin{array}{cc} u(\theta ) & t(\theta )\\ t(\theta ) & v(\theta ) \end{array}\right] \end{array}$$

where

$$t\left(\theta \right)=\sin\theta \cdot \cos\theta \cdot \left({\left({}^{M}y_{i}\right)}^{2}-{\left({}^{M}x_{i}\right)}^{2}\right)+{}^{M}x_{i}\cdot {}^{M}y_{i}\cdot \left({\left(\sin\theta \right)}^{2}-{\left(\cos\theta \right)}^{2}\right)$$

$$u\left(\theta \right)={\left(-\sin\theta \cdot^{M}x_{i}-\cos\theta \cdot^{M}y_{i}\right)}^{2}$$

$$v\left(\theta \right)=(\cos\theta \cdot^{M}x_{i}-\sin\theta \cdot^{M}y_{i}{)}^{2}$$

From the above calculation, one can deduce that the uncertainty of the heading angle θ is not uniformly propagated to the lane grid cells. The uncertainty of one cell in the *x* direction increases with regards to the distance augmentation to the host vehicle. In the *y* direction, the uncertainty of one cell increases with regards to the *x* coordinate augmentation. In conclusion, the uncertainty of one cell caused by heading error increases with regards to the *x* direction.

Generally speaking, if we now denote the uncertainty of cell *i* in the global frame $Var{(}^{O}X_{i})$, let $f{(}^{O}x_{M},{\;}^{O}y_{M},\;\theta )$ denotes the transformation Equation (2). Thus

(5) $$g_{i}\left(x,\;y\right)=\;Var{(}^{O}X_{i})\;=\left[\frac{\delta f}{\delta^{O}X_{M}}\right]\cdot \;P_{{}^{O}X_{M}}\;\cdot {\left[\frac{\delta f}{\delta^{O}X_{M}}\right]}^{T}$$

where $P_{{}^{O}X_{i}}$ represents the covariance matrix of the 2D position ${}^{O}X_{M}$ =${(}^{O}x_{M},{\;}^{O}y_{M},\;\theta )$, and

$$\left[\frac{\delta f}{\delta {}^{O}X_{M}}\right]={\left[\begin{array}{c} \frac{\delta f}{\delta^{O}x_{M}}\\ \frac{\delta f}{\delta^{O}y_{M}}\\ \frac{\delta f}{\delta \theta } \end{array}\right]}^{T}$$

#### 2.3.2. Probabilistic Approach to Tackle Grid Cell Uncertainty

In this section, a probabilistic method to compute the belief for each grid cell is proposed. The lane belief distribution constructed in Section 2.2 is used, let denote B(i,A) the probability that lane *i* to be in state *A*. Herein, the probability of lane *i* to be in state *A* is represented as $P\left(S_{l_{i}}=A\right)=B\left(i,\;A\right)$.

Probabilities are for single states, i.e., probability can only be assigned to one of the states in Ω={Ego,Accessible,Forbidden}. Since we have the probability distribution of the lanes, in order to have probability distribution for a single cell, we need to find out where the cell is localized.

Let us take the red cell *i* in Figure 8 as an example. We define two properties for each cell *i* in the grid, respectively $L_{i}$ which indicates the lane index of this cell and $S_{i}$ for the state of this cell. Thus, $L_{i}\in \left(1,2,\;...\;nl\right)$ where nl represents the number of lanes over the road, and $S_{i}\in \left\{Ego,\;Accessible,\;Forbidden\right\}$.

Thus, the probability of cell *i* being in lane *k* can be calculated as

(6) $$P\left(L_{i}=k\right)=\underset{\left(x,y\in Lane_{k}\right)}{\;\int \int \;}g_{i}\left(x,\;y\right)d_{x}d_{y}.$$

Remember that $g_{i}\left(x,y\right)$ is the extrapolated uncertainty in the global frame, x,y being the location in this frame. Using the total probability law, one can compute the probability of each state for the cell. For instance, the probability of the state *A* for cell *i* can be calculated as:

$$P\left(S_{i}=A\right)=\sum_{k=1}^{nl}P\left(S_{i}=A\;|\;L_{i}=k\right)\cdot P\left(L_{i}=k\right).$$

We need to compute each part of this equation to get the final result. $P(L_{i}=k)$ has been already tackled previously. Now, the problem resides in computing the first part $P(S_{i}=A\;|\;L_{i}=k)$. To go through this problem, we suppose the state for lane *k* is denoted as $S_{l_{k}}$. In addition, we can develop the first part into

$$P\left(S_{i}=A\;|\;L_{i}=k\right)=P\left(S_{i}=A\;|\;S_{i}=S_{l_{k}}\right)$$

because we know if one cell lies in lane *k*, then it has the same state as lane *k*.

Furthermore,

$$P\left(S_{i}=A\;|\;S_{i}=S_{l_{k}}\right)=P\left(S_{l_{k}}=A\right)=B\left(k,\;A\right).$$

Remember that B(k,A) is the lane distribution. Finally, the probability of the state A for cell i can be calculated as:

$$P\left(S_{i}=A\right)=\sum_{k=1}^{nl}B\left(k,\;A\right)\;\cdot \;\underset{\left(x,y\in Lane_{k}\right)}{\;\int \int \;}g_{i}\left(x,\;y\right)d_{x}d_{y}.$$

This process is repeated for every cell in the grid.

#### 2.3.3. Evidential Approach to Tackle Grid Cell Uncertainty

In the continuity of the works on evidential occupation grids, we propose the Dempster–Shafer’s theory to deal with uncertainties. The frame of discernment

$$\Omega =\left\{Ego,\;Accessible,\;Forbidden\right\},$$

corresponds to the lane states. These singletons are mutually exclusive.

The power set is thus defined as:

(7) $$\begin{array}{cc} 2^{\Omega } & =\{\emptyset ,\;Ego,\;Accessible,\;Forbidden,\;\{Ego,\;Accessible\},\ldots \end{array}$$

(8) $$\begin{array}{cc} \; & \;\;\;\;\;\;\;\;\;\{Ego,\;Forbidden\},\;\{Accessible,\;Forbidden\},\;\Omega \} \end{array}$$

An advantage of the evidential representation of the lane information using the Dempster–Shafer’s theory is that we may attribute mass of evidence to any subset of the frame of discernment, for example {Ego,Accessible}. This is the case when we are not able to tell whether the mass should be assigned to Ego or Accessible. For our problem, we can take advantage of this property to model the lane marking space. Since the lane markings are the boundaries of lanes, if the two lanes which are separated by one lane marking have two different lane states, then we will assign the mass to the union of the two states.

Moreover, the mass can also be put in Ω, which indicates the level of ignorance.

The meaning of each proposition is thus detailed below:

1. Ego indicates the cells which are inside the current occupied lane of host vehicle (Ego lane).
2. Accessible indicates the cells which are inside the permitted lanes by road rules but not the current occupied lane of host vehicle (i.e., Accessible lane).
3. Forbidden indicates the cells which are not inside any permitted lane (Forbidden Lane).
4. {Ego,Accessible} indicates the cells whose states can be both Ego and Accessible, but one cannot determine which. Normally this proposition describes the lane markings separating the Ego lane and Accessible lane.
5. {Ego,Forbidden} indicates the cells whose states can be both Ego and Forbidden, but one cannot determine which. Normally this proposition describes the lane markings separating the Ego lane and Forbidden lane.
6. {Accessible,Forbidden} indicates the cells whose states can be both Accessible and Forbidden, but one cannot determine which. Normally this proposition describes the lane markings separating the Accessible lane and Forbidden lane.
7. Ω indicates ignorance about the state of the cell (Unknowncell).
8. ∅ indicates that no proposition fits the cell.

To build now the mass function m() for grid cells, we use the computed position uncertainty and lane belief distribution. Each cell in the lane grid can belong to any of the lanes defined in Section 2.2. If one cell lies inside one lane, then it should have the same mass distribution as the lane.

We have

$$m^{i}=m_{k},\;\;if\;C_{i}\;\in Lane_{k}$$

in which $m^{i}$ is the mass distribution of cell *i* ($C_{i}$), $m_{k}$ is the mass distribution of $Lane_{k}$ constructed in Section 2.2 such as $m_{k}\left(A\right)=B\left(k,\;A\right)$, where *k* represents the lane index and *A* represents the lane state.

However, to which lane belongs the cell is not deterministic, due to the position uncertainty. Based on the position uncertainty computed in Section 2.2.1, a confidence level can be defined:

(9) $$\alpha_{k}^{i}=\underset{\left(x,y\in Lane_{k}\right)}{\;\int \int \;}g_{i}\left(x,\;y\right)d_{x}d_{y}.$$

This confidence level is applied to discount the mass distribution of lane *k*, the result is considered to be a source of information provided by this lane. Specifically, for lane *k*, we have its mass distribution $m_{k}$, the confidence level $\alpha_{k}^{i}$, then the information provided by this lane is computed as:

$$\begin{array}{c} m_{k}^{i}\left(A\right)=\alpha_{k}^{i}\;\cdot \;m_{k}\left(A\right),\;A\neq \Omega \\ m_{k}^{i}\left(\Omega \right)=\alpha_{k}^{i}\;\cdot \;m_{k}\left(\Omega \right)+1-\alpha_{k}^{i} \end{array}$$

The process is repeated using each lane, thus each lane is considered as one source of information providing a mass distribution. Combining all the information provided by all the lanes, we can then have the mass distribution for one cell.

Thus, the mass distribution for cell *i* can be computed by

(10) $$m^{i}={⊚}_{{}_{k}}m_{k}^{i},\;\;k=1,2,\;\ldots \;,nl$$

in which *k* is the lane index, nl is the number of lanes.

We use the operator ⊚ proposed in [33] defined as follows:

$$\left\{\begin{array}{cc} \begin{array}{c} \left(m_{1}⊚m_{2}\right)\left(A\right)=\sum_{B\cap C=A\neq \emptyset }m_{1}\left(B\right)\cdot m_{2}\left(C\right)\\ \left(m_{1}⊚m_{2}\right)\left(A\right)=\sum_{B\cap C=\emptyset ,\;B\cup C=A}m_{1}\left(B\right)\cdot m_{2}\left(C\right) \end{array} & A,\;B,\;C\subset \Omega \end{array}\right.$$

The specialty of this operator is that the conflicting mass will be put into union of propositions. The reason for this transfer of mass is that the conflicting mass in one cell origins from position uncertainty and indicates multi-states information which gives hint about lane boundaries. Only the cells close to the lane boundaries can have large conflicting information if the position uncertainty is small enough.

## 3. Combination of Occupancy and Semantic Information in Grids

In this section, we propose to combine the Semantic lane grid with an Occupancy grid to build a new grid called Perception grid that represents both static and dynamic local environment of a vehicle. The process to build the Occupancy grid is detailed in [34]. The space was noted either Occupied or Free of obstacle.

### 3.1. Defining a Common Frame of Discernment

The occupancy grids are defined by the frame of discernment $\Omega_{O}=\left\{O,\;F\right\}$. *O* for Occupied state and *F* for Free state. For semantic lane grid, the frame of discernment is $\Omega_{L}=\left\{Ego,\;Accessible,\;Forbidden\right\}$. To combine these two grids defined in different frames of discernment, one needs to define a common frame of discernment to enable information fusion. Herein, the common frame of discernment is defined as

$$\Omega_{C}=\left\{\text{Ego\_Free},\text{Accessible\_Free},\text{Forbidden\_Free},\text{Non\_Navigable}\right\}$$

This definition has the advantage of maintaining the Free state information in different lanes. The Non_Navigable information is a combination of the Occupied information as well as the non-accessible lane information.

The meanings of the singletons are as follows:

- Ego_Free represents Free cells located in the Ego lane.
- Accessible_Free represents Free cells located in an Accessible lane.
- Forbidden_Free represents Free cells located in a Forbidden lane.
- Non_Navigable represents Occupied cells, regardless of their location.

This defined common frame of discernment $\Omega_{C}$ is in fact a refinement of both $\Omega_{O}$ and $\Omega_{L}$. The mapping of the refinement is defined as:

From $\Omega_{O}$ to $\Omega_{C}$:

$$\rho_{OC}:\;2^{\Omega_{O}}\to 2^{\Omega_{C}}$$

{O}↦{Non_Navigable}

{F}↦{Ego_Free,Accessible_Free,Forbidden_Free}

$$\Omega_{O}\;\;\mapsto \Omega_{C}$$

From $\Omega_{L}$ to $\Omega_{C}$:

$$\rho_{LC}:\;2^{\Omega_{L}}\to 2^{\Omega_{C}}$$

{Ego}↦{Ego_Free,Non_Navigable}

{Accessible}↦{Accessible_Free,Non_Navigable}

{Forbidden}↦{Forbidden_Free,Non_Navigable}

{Ego,Accessible}↦{Ego_Free,Accessible_Free,Non_Navigable}

{Ego,Forbidden}↦{Ego_Free,Forbidden_Free,Non_Navigable}

{Accessible,Forbidden}↦{Accessible_Free,Forbidden_Free,Non_Navigable}

$$\Omega_{L}\;\;\mapsto \Omega_{C}$$

Using the above refinement, the mass transfers are performed as follows:

$$m^{\Omega_{O}↑\Omega_{C}}\left(\rho_{OC}\left(A\right)\right)=m^{\Omega_{O}}\left(A\right)\;\forall A\subseteq \Omega_{O}$$

$$m^{\Omega_{L}↑\Omega_{C}}\left(\rho_{LC}\left(B\right)\right)=m^{\Omega_{L}}\left(B\right)\;\forall B\subseteq \Omega_{L}$$

Using these formulas, the information from the occupancy grids and the lane grids can be transferred into the same frame of discernment $\Omega_{C}$.

### 3.2. Combination of the Two Grids by Fusion

A this stage, the two sources of information (the grids) are defined in the same common frame of discernment. We suppose that the grids have the same resolution and the same size. A combination must be performed to get a final grid. It can be done by fusing the two grids based on the Dempster’s rule, since these two sources of information are both reliable and independent.

The fusion is performed for every cell as:

$$m^{\Omega_{C}}=m^{\Omega_{O}↑\Omega_{C}}⊕m^{\Omega_{L}↑\Omega_{C}}$$

where $m^{\Omega_{C}}$ represents the mass function of the cell of the combined grid.

One might be interested to investigate the details of the fusion process, since the Dempster’s rule implies a normalization process which redistributes the conflict information into the non-conflict states according to their mass distribution. However, the common frame $\Omega_{C}$ was designed to tackle this problem. In fact, after the two refinement processes, there exists no conflict mass in these two sources of information.

## 4. Real Road Experiments and Results

### 4.1. Real Road Experiments

**Figure 9:**
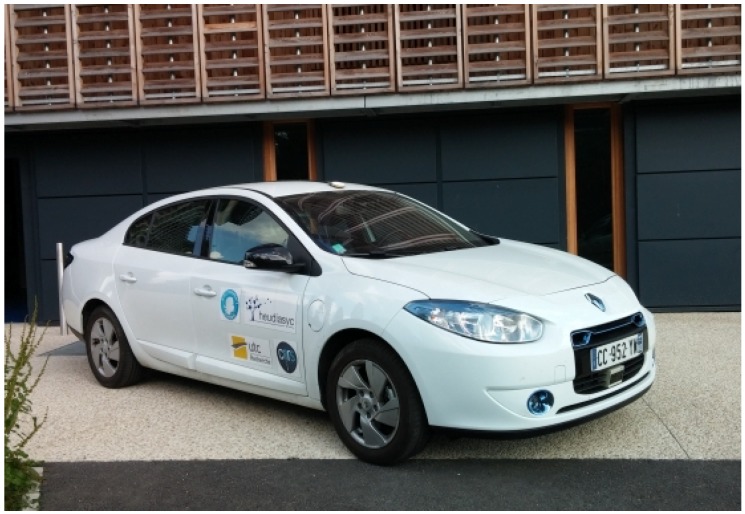
The experimental vehicle.

Real road experiments have been done with an experimental vehicle of the Heudiasyc Laboratory. The vehicle is shown in Figure 9 and the sensor configuration is similar to [35]. For semantic lane grid, the inputs of the algorithm are the map and the vehicle pose with its covariance matrix (provided by a localization system implementing a Kalman filter). We have used a high-definition map with negligible error level. In the map, the road is explicitly described with lane information, including lane markings and road boundary. The lane markings are distinguished in the map with different attributes which are important to determine the lane state. The vehicle pose comes from a GPS system with RTK corrections. This system can provide positioning with high accuracy in RTK-fixed operation mode.

We have used a LiDAR (SICK LDMRS) installed in the front of the vehicle to construct the occupancy grid. During the experiments, the LiDAR was triggered by the GPS receiver.

To qualitatively evaluate our result, we have adopted the approach proposed in [36]. A wide-angle scene camera has been installed behind the windshield during the acquisition process in the experiment. The camera was also synchronized with the GPS receiver. The constructed lane grid is projected on the scene image captured by the camera to provide a qualitative evaluation indicator. This method enables the evaluation of the correspondence of the grids with regards to the observed scene. The calibration between the camera and the GPS antenna has been performed off-line. The retro-projection process consists in computing the 2D image coordinates corresponding to each grid cell vertex.

**Figure 10:**
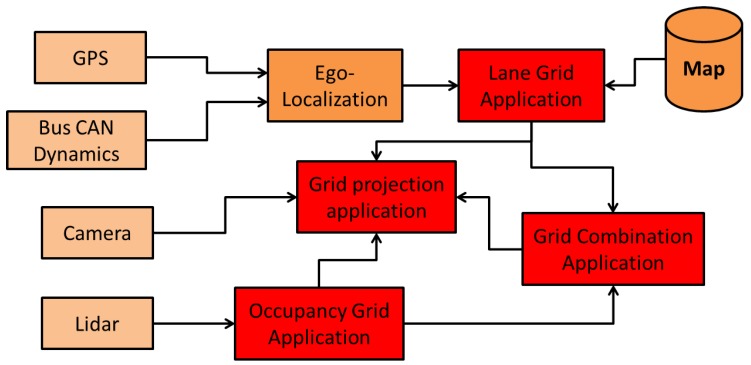
Software implementation in red.

The system has been implemented on C++ with a Linux computer. Figure 10 shows the system and its 9 components. The inputs are a GPS receiver, a CAN bus gateway of the vehicle to access to the speeds, a LiDAR driver and a camera for visualization. The output of a GPS receiver and CAN bus data (wheel speed and yaw rate) are used in the ego-localization process to estimate the absolute pose. The semantic lane grid is constructed in the Lane Grid Application component, where the estimated pose and the prior map are taken as inputs. The evaluation process takes place in the Grid projection application component. The lane grid is projected on the image coming from the scene camera. The component LiDAR acquires the data from the SICK LiDAR which is sent to the “Occupancy grid Application” to construct the occupancy grid in real time. This occupancy grid serves as input to the “Grid combination Application” with the lane grid from the “Lane Grid Application”. A qualitative evaluation is performed by projecting the combination grid onto the scene images.

We show grids of 40×16 m in length and width. The cells are with a size of (0.1×0.1) m. In this part, grids constructed with two different levels of pose uncertainty are given. For notation purpose, we herein use $\left(\sigma_{x},\;\sigma_{y}\;,\sigma_{\theta }\right)$ as the 2D pose uncertainty. The grids are shown by visualization of a RGB image. The RGB image enables reflection of the belief level by the RGB color channel brightness. Brighter color reflects higher belief level.

### 4.2. Experimental Results

**Figure 11:**
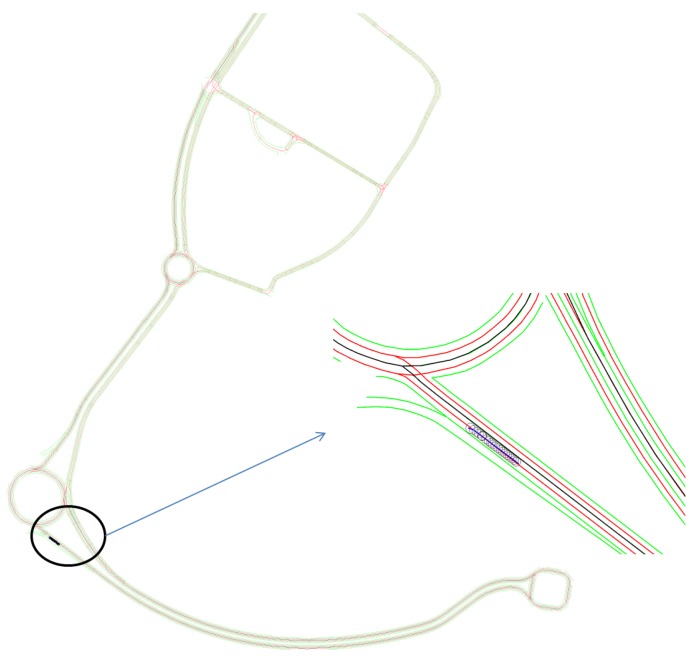
Projection of the vehicle position on the map. This map of Compiègne (France) has been charted by a professional in the framework of the Robotex project.

In this section, semantic lane grid results of both (probabilistic and evidential) approaches are given. For the purpose of demonstration, a projection of the host vehicle’s position on a prior map is displayed in Figure 11. The bird view of the map is given with a zoom-out at the region where the host vehicle is located. In this situation, the host vehicle is running on a three-lane road. The vehicle is in the middle lane, whereas the lane on the right is for the purpose of entering the road, so this lane is not accessible. The lane on the left is a parallel lane with the same orientation than the current occupied lane, thus this lane is accessible.

#### 4.2.1. Probabilistic Lane Grid Result

**Figure 12:**
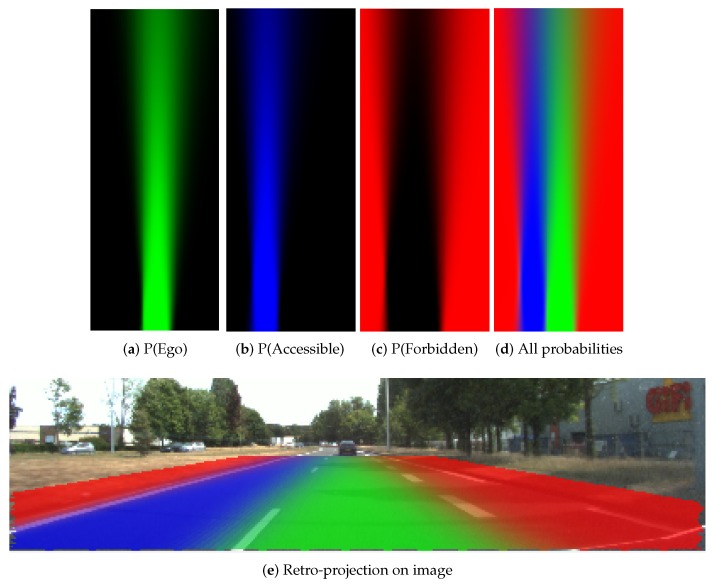
Probabilistic grids with small uncertainty $\left(\sigma_{x}=0.2\;m,\;\sigma_{y}=0.3\;m\;,\sigma_{\theta }=0.1\;\mathrm{radians}\right)$.

**Figure 13:**
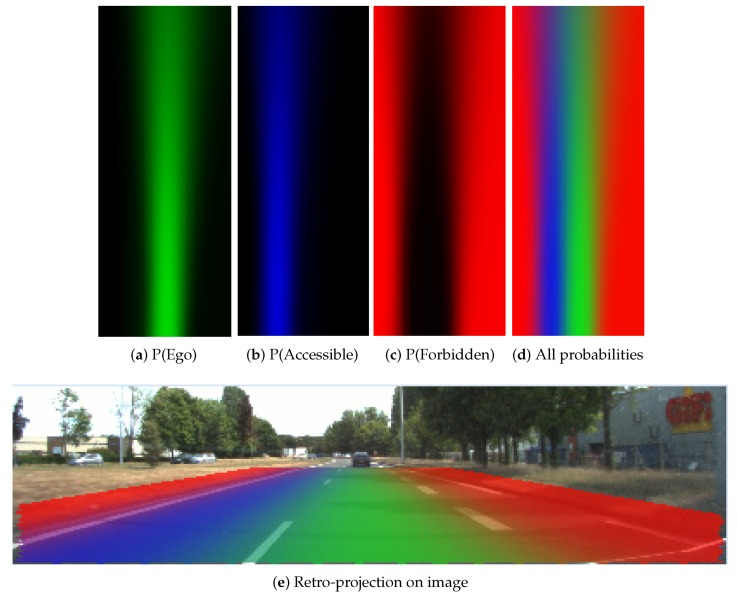
Probabilistic grids with large uncertainty $\left(\sigma_{x}=0.9\;m,\;\sigma_{y}=1.1\;m\;,\sigma_{\theta }=0.1\;\mathrm{radians}\right)$.

In Figure 12, the probabilistic grids are shown with the pose uncertainty $\left(\sigma_{x}=0.2\;m,\sigma_{y}=0.3\;m\;,\sigma_{\theta }=0.1\;\mathrm{radians}\right)$, and in Figure 13 shows the results with a larger pose uncertainty $\left(\sigma_{x}=0.9\;m,\;\sigma_{y}=1.1\;m\;,\sigma_{\theta }=0.1\;\mathrm{radians}\right)$. Corresponding to each uncertainty, the probability distribution of each lane state is shown separately. The combination of all states is also shown, as well as the retro-projection of the distribution on the scene image.

From these results, one can clearly remark that cells at farther distance have lower probability level according to the decreasing color level in the grid, especially in Figure 13. The probability distribution extends to adjacent lanes, which means a dispersed probability. This is the consequence of uncertainty propagation. The combined probability shown in Figure 12d and Figure 13d further reflect this phenomenon. The lane cells that are located close to the host vehicle contain single state probability, whereas the probability distribution of the cells at farther distance can become very ambiguous and dispersed.

In Figure 12e and Figure 13e, the retro-projection of the constructed grids on the images provides a qualitative view of evaluation. The features reflecting the lanes are valid based on the correspondence between the grids and the image spaces.

#### 4.2.2. Evidential Lane Grid Result

**Figure 14:**
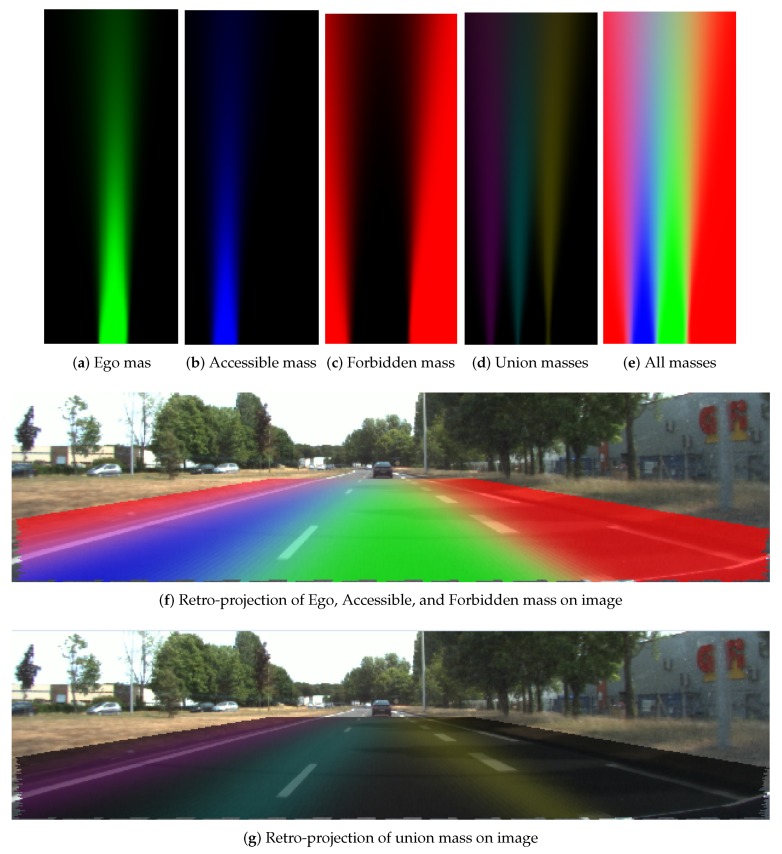
Resultant grids of the evidential approach, small position uncertainty $\left(\sigma_{x}=0.2\;m,\;\sigma_{y}=0.3\;m\;,\sigma_{\theta }=0.1\;\mathrm{radians}\right)$.

**Figure 15:**
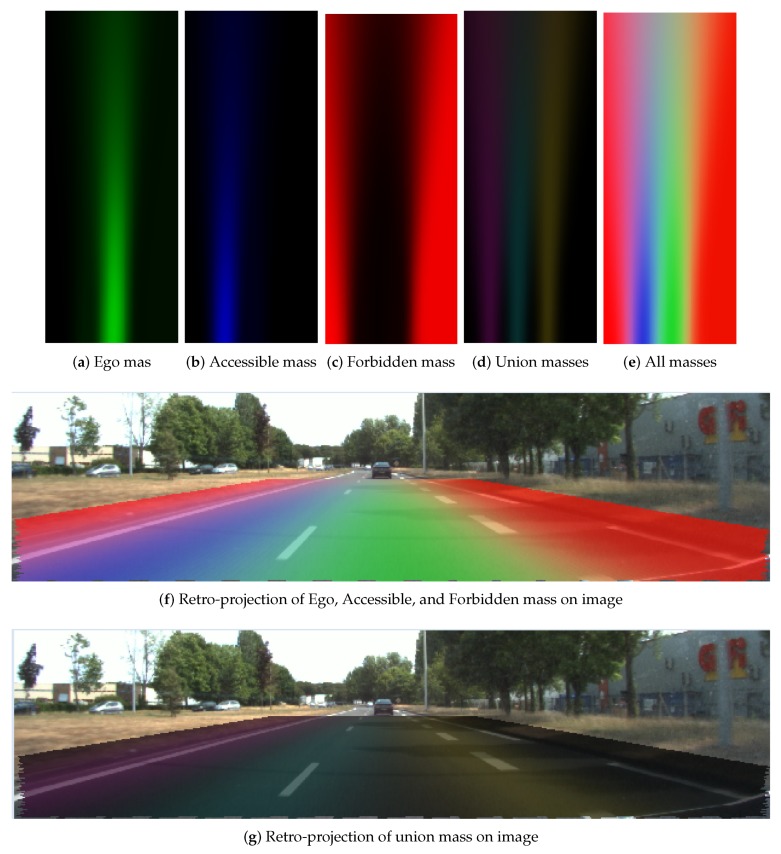
Resultant grids of evidential approach, large position uncertainty $\left(\sigma_{x}=0.9\;m,\;\sigma_{y}=1.1\;m,\sigma_{\theta }=0.1\;\mathrm{radians}\right)$.

Figure 14 and Figure 15 display respectively the resultant lane grids of the evidential approach with the small position uncertainty $\left(\sigma_{x}=0.2\;m,\;\sigma_{y}=0.3\;m\;,\sigma_{\theta }=0.1\;\mathrm{radians}\right)$ and the large one $\left(\sigma_{x}=0.9\;m,\;\sigma_{y}=1.1\;m,\sigma_{\theta }=0.1\;\mathrm{radians}\right)$. In these two figures, the mass distributions are shown in the format of RGB images. From the color variance in the figure, one can clearly remark that grid cells have different mass level over distance change. Cells that are closer to the host vehicle tend to have higher level of mass in each state. This is due to the uncertainty of heading angle causing the farther cells to have larger uncertainty. The mass in the union of the states also demonstrate this effect. The mass in the union states are shown in Figure 14d and Figure 15d, and is displayed by the combination of colors: yellow, cyan and magenta colors represent respectively the mass in {Ego,Forbidden}, {Ego,Accessible} and {Accessible,Forbidden}. Moreover, one can remark from the retro-projection that the union of the masses focus mainly on the cells that are on the markings or close to the markings since the proposed operator takes full advantage of the property that the union of the masses should be assigned to union of the states that cannot be separated.

The difference between the resultant lane grids of the two different uncertainties is obvious. A larger pose uncertainty level results in less mass in the singleton states and more mass in the union states. The Ego mass level in Figure 14a is clearly higher than in Figure 15a, and in Figure 15d clearly the union mass area (left to right: {Accessible,Forbidden}, {Ego,Accessible}, {Ego,Forbidden}) is larger than in Figure 14d.

In Figure 14f and Figure 15f, the retro-projections of Ego, Accessible, and Forbidden mass distributions are displayed. One can see that the lane information is correctly integrated in the grid. One should keep in mind that the road rules are implied in the cell states by different masses supporting each state. Figure 14g and Figure 15g show the retro-projection of the {Ego, Accessible}, {Ego, Forbidden} and {Accessible,Forbidden} masses. One can see these masses are concentrated over the markings. This is the advantage of the adopted operator.

The system has been tested of many roads and the reader can watch the following video online to get a better idea (https://youtu.be/0fJp-d4K75s).

#### 4.2.3. Comparison between Probabilistic and Evidential Lane Grids

**Figure 16:**
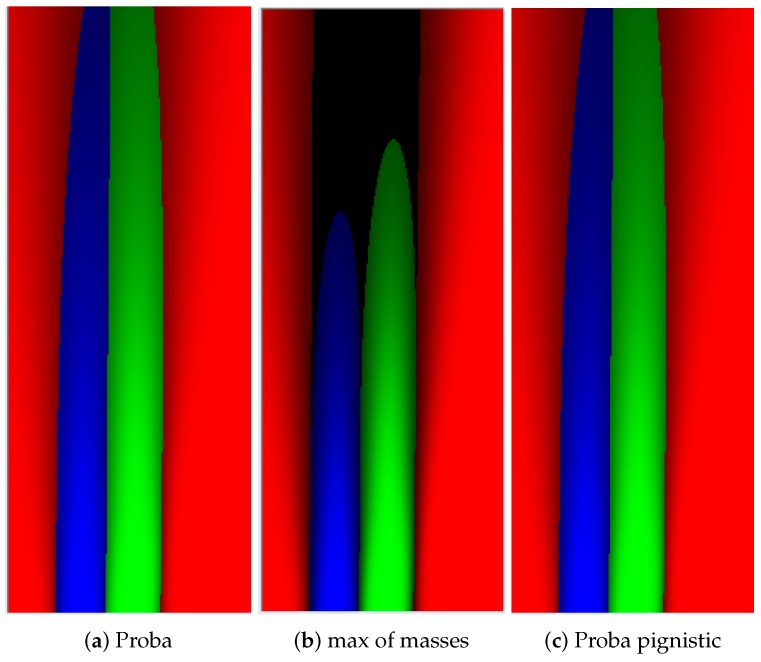
Decision grids used for path planning for instance.

Compared to probabilistic approach, the evidential approach provides more flexible way to handle uncertainty. It provides possibility to put belief into union states if, for example, the belief in each single state is not clear. The Unknown mass can explicitly quantify ignorance, which avoids putting prior information to the cases where no data support any state. In the evidential approach, the pignistic probability [37] can be adopted to transform masses to probabilities. In Figure 16a,c, we herein compare the pignistic probability grid and probabilistic decision grid. The ratio of identical decision between these two decision grids is 99.992%, which means the approach to handle uncertainty by evidential theory is valid.

Figure 16b illustrates the main difference of these two methods. This is the decision grid deduced by the maximum of evidence masses. The dark space is marked Unknown which means no decision of state is made over these spaces, because not enough information is provided. This is an important advantage over the probabilistic approach as this evidential decision grid enables the avoidance of risky trajectory with insufficient information provided.

#### 4.2.4. Information Discussion

In this section, we give an analysis of the resultant lane grids by introducing the Specificity and Entropy. Together they give an evaluative view about the quality of the mass distribution of each cell in the lane grid. Average Specificity and Entropy values are calculated for eachlane grid [38]. According to the definition of specificity and entropy, an informative and non-ambiguous mass function should have a high degree of specificity and a low degree of entropy.

##### Average Entropy and Specificity Variation Regarding to Position Uncertainty

Let study how these two measures evolve with regards to the position uncertainty. The study is simply conducted by adding random noises to a specific position of the host vehicle. This is a Monte-Carlo method. Every uncertainty level is sampled 1000 times and corresponding lane grids are constructed. The average entropy and specificity value are computed in each case.

**Figure 17:**
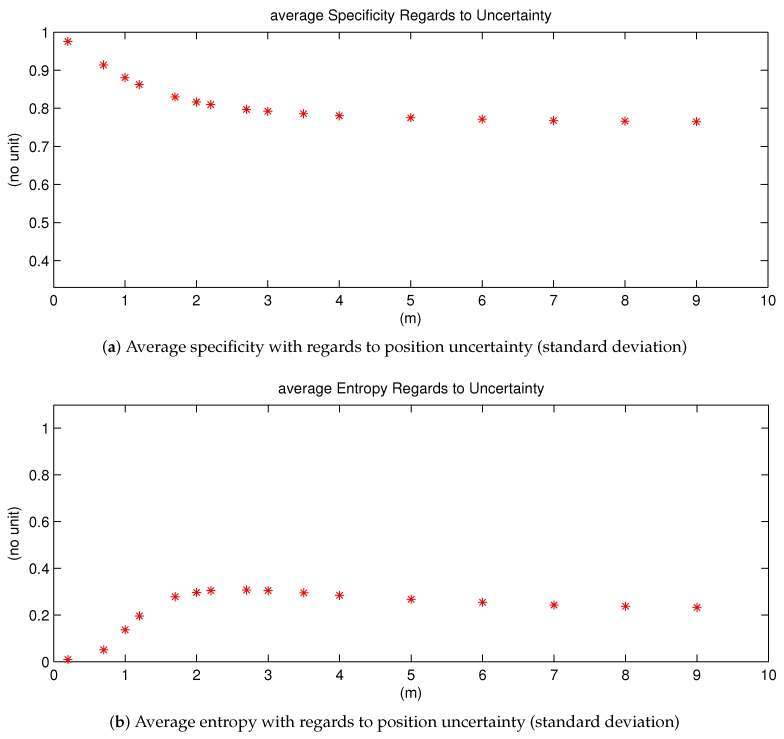
Average specificity and average entropy with regards to position uncertainty variation.

In Figure 17, simulation results are shown, the x-axis of both images represent uncertainty variation. Figure 17a shows the average specificity measure. One can remark that if the uncertainty level augments, the specificity measure gradually decreases, and at last converges to a constant value. This behavior demonstrates the fact that the specificity value should be smaller with a larger uncertainty level. Furthermore, we can deduce that if the position uncertainty becomes too large up to an inapplicable level, the average specificity value converges to a constant level, which is larger than the minimal value $\frac{1}{3}$. This is reasonable because there will be always certain quantity of mass in singleton states.

Similar to the Specificity measure, the Entropy measure shown in Figure 17b eventually converges to a stable value as well. The explication is the same as for the Specificity measure. However, one can remark a difference: its value augments at first, then gradually decreases to the stable value, with regards to uncertainty level. With no uncertainty, the entropy value is 0 at first, then increasing uncertainty leads to dissonant mass thus larger entropy value. However, if the uncertainty continues to increase, according to the discounting process, more mass thus goes to ignorance and the mass in ignorance does not conflict with other states. Thus, finally the entropy measure goes down until convergence level.

The behaviors of the specificity and entropy demonstrate that the constructed lane grids become less informative with larger position uncertainty. This comportment means that the provided information from the constructed lane grids is consistent with input uncertainty. Thus, we can conclude that the proposed approach has well tackled the uncertainty.

### 4.3. Combination Grid Results

**Figure 18:**
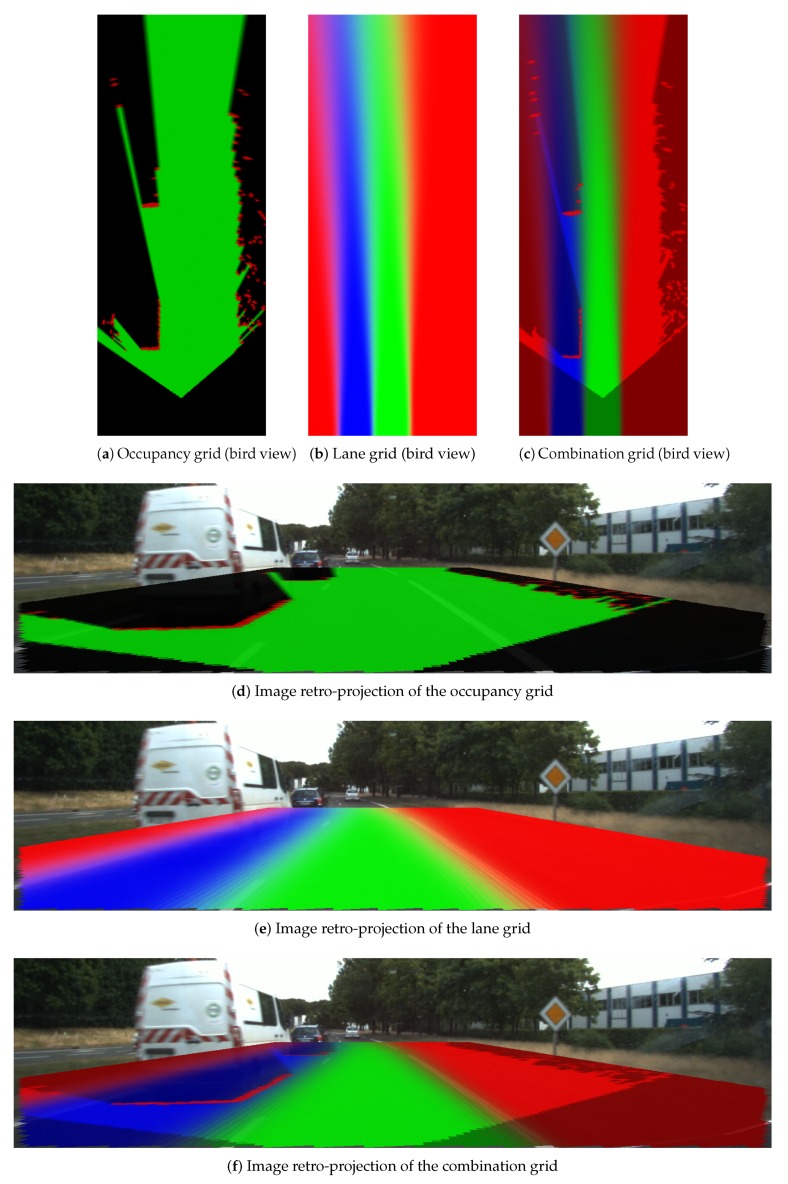
Results for scene one. In occupancy grids, red means Occupied, green Free, dark Unknown. In lane grids, red Forbidden, green Ego, blue Accessible. In combination grids, red Non_Navigable plus Forbidden_Free, green Ego_Free, Blue Accessible_Free.

**Figure 19:**
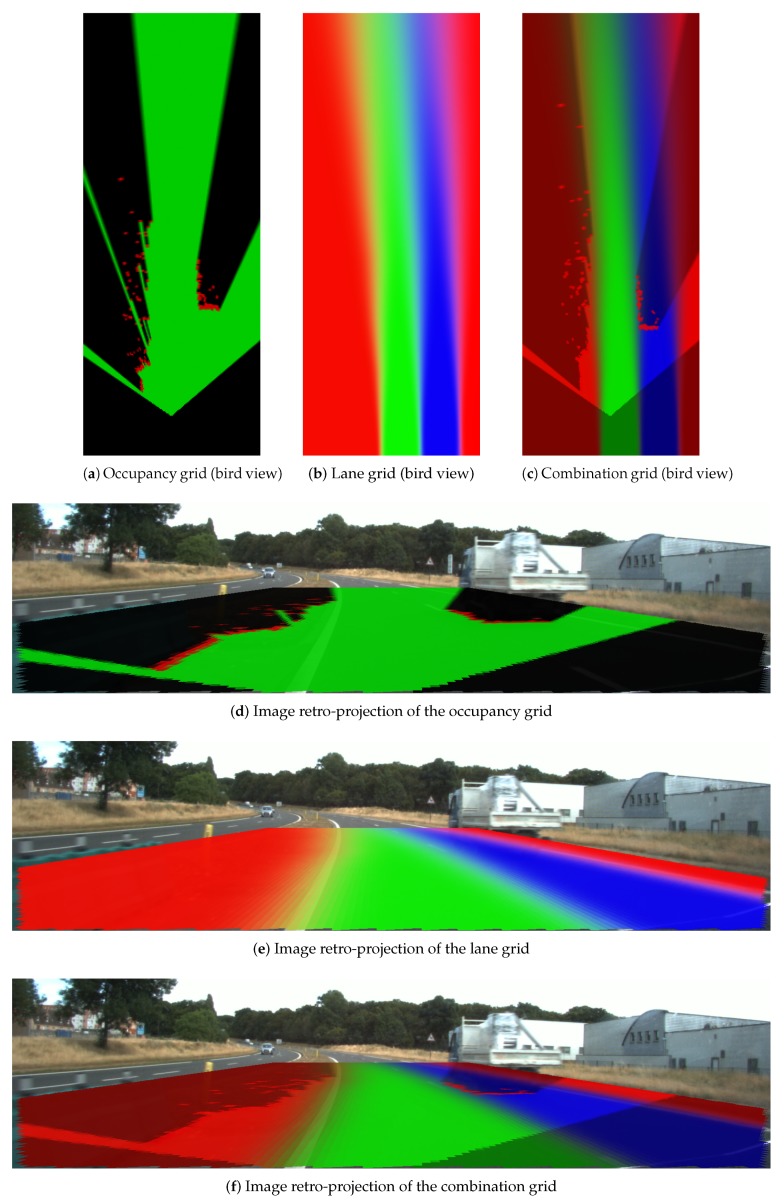
Result scene two. Same color code as in Figure 18.

**Figure 20:**
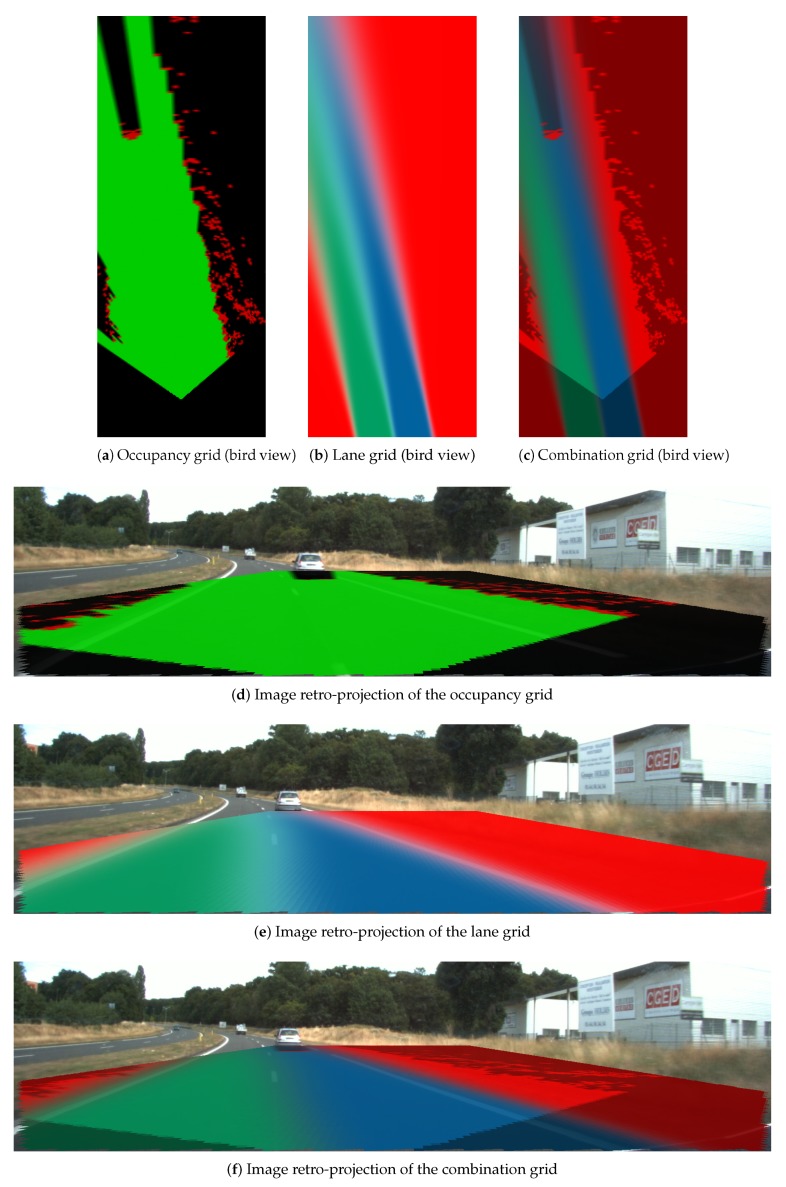
Result scene three when the vehicle is doing a lane change (from left to right). Same color code as in Figure 18.

Figure 18, Figure 19 and Figure 20 display three particular results. Bird views of the occupancy grids, lane grids, and combination grids are shown in the top row. They are front-looking grids, the vehicle being located at the bottom on the grids. More precisely, the grids have their origin exactly located at the middle of the rear axis of the car (and not at the front bumper as often made). This is to make them usable by a path planer.

The projection of the respective grids on the scene image are shown afterwards for a qualitative evaluation. Herein green, red, dark and blue colors are applied in these grids. The color code is identical as the one introduced for occupancy grids (green: free, red occupied) and lane grids. For the combination grid, since the frame of discernment is defined as $\Omega_{C}$ = {Ego_Free,Accessible_Free, Forbidden_Free,Non_Navigable}, we use for display the green color which represents the Ego_Free level, the blue color for the Accessible_Free level, while the red color represents the sum of Forbidden_Free and Non_Navigable levels. The state level shown in the combination grids are the pignistic probabilities calculated after the fusion process.

From the occupancy grid, one can remark from the projection on the scene image that the obstacle vehicle is correctly detected (even if there is a small calibration error on the reprojection). Moreover, the LiDAR is installed in the front of the host vehicle and this is the origin of the constructed occupancy grid. However, based on the approach introduced in Section 2, the lane grids are constructed on local coordinates defined at the center of the vehicle’s rear axle, i.e., the origin *M*. Herein, to compensate for this coordinate difference, a translation is performed for the occupancy grid. The resulting dark region at the bottom of the occupancy grid shows this translation. This area is an unperceived area (a dead zone), so total ignorance is assigned.

The lane grid shown in Figure 18 is constructed by taking into account the pose uncertainty $\left(\sigma_{x}=0.2\;m,\;\sigma_{y}=0.3\;m\;,\sigma_{\theta }=0.05\;\mathrm{radians}\right)$. The effect of the uncertainty propagation can still be seen in the grid even if the angular uncertainty is small. The projection on the scene image shows that the semantic lane information is correctly modeled in the grid. One can also remark that the lanes (which are not straight in this experiment) are well characterized over a long distance ahead. In this particular situation, the characterization of the navigability of the lanes would be difficult to do with only on-board sensors like cameras. The advantage of using a localization system with a map is here clearly highlighted.

Figure 20 displays the result when the host vehicle was performing a lane changing from the left lane to the right lane. This lane change is particularly visible in the lane grid since the lanes are rotated on the left. In this kind of situation, one can remark from the mass level in the lane grid that the Ego and Accessible states are uncertain (Green and blue color are not bright compared to the other two scene results), which is conform to the fact that the vehicle is running across to the dashed lane marking which separates the two lanes. The determination of the lane states in this kind of transition stage is very ambiguous and difficult to perform and the behavior of the perception system perfectly fits with reality.

Within the combination grid, the perception system handles obstacle information as well as semantic lane information in a unique representation of the world which is important for a path planning module as said before. Thus, we have kept the Free information in both the Ego lane and Accessible lane. The obstacle information is drawn in red along with all the cells in the Forbidden region.

Instead of storing the obstacle information and the semantic lane information in two separate grids, the combination process manages these two sources of information into a uniform frame which facilitates path planning afterwards. For the reinforcement of the grid application, the additional Ego_Free information makes it possible to find the path all along the ego lane for lane-keeping application, whereas the Accessible_Free information can serve as a second choice when the ego lane is blocked.

The system has been tested on many roads and the reader can watch the video online (https://youtu.be/0F078KJkSRo) of a 1.5 km long trial to evaluate the performance of the method.

### 4.4. Processing Time

The average time of execution is computed corresponding to the same grid resolutions as before. Each step in the approach is timed and the average time consumption is shown in Table 1. The grid size is set to be (40 × 16 m) for all the shown results.

One can remark that the refinement and the fusion process add little processing time.

At 0.1 m resolution, we need more than a half of second to construct the combination grid, which is not compatible with a real time implementation. However, at 0.4 m, the average time consumption decreases to less than 0.1 s. This normally can meet the requirement of real-time applications. In fact, the shown results can be optimized through parallel programming, which has not been implemented in the current system.

## 5. Conclusions

A new grid-based approach to characterize the lane information, integrating semantic road rule into the grid cells has been proposed. Based on the Dempster–Shafer theory, a road rule is interpreted as semantic information of accessibility contained in the Semantic lane Grid. The Ego, Accessible and Forbidden propositions defined in the frame of discernment characterizes the road space into meaningful parts, and the boundaries of these meaningful parts contains the uncertain mass. We have proposed a multi-hypothesis model to take into account the host vehicle’s pose uncertainty relative to the road. Simulation results as well as real road experimental results have been reported.

A Dempster fusion process has been introduced to combine obstacle information and semantic lane information. They characterize the state of the same space with complementary aspects. The fusion process provides a more complete representation of the environment in a unique perception grid keeping the key information for path planning applications with a negligible additional processing time.

The results show interesting perspective in the domain of intelligent vehicle perception and navigation especially when HD maps will be available worldwide. Applying this resulting grid as an input to a path planner would be an interesting application of this work. New level of information can be added as an extension of this work, like emergency region, public transport region, etc. Moreover, an evaluation of this approach with a less accurate map would be interesting, since maps are often affected by errors. On the other hand, the hypothesis of an accurate prior map is not realistic, which is the main limitation of the proposed approach.

## Figures and Tables

**Table 1:** Average execution time in seconds used by each procedure in the evidential grid construction. The grid size was set at (40 × 16) m. Tested on PC of processor Inter(R) Core(TM) i7, CPU @ 2.30GHz. RAM 8Go.

Resolution (meters)	Occupancy Grid	Lane Grid	Combination	Total Time
0.1	0.0311	0.4933	0.0066	0.5310
0.2	0.0206	0.173	0.00147	0.1951
0.4	0.0160	0.0662	0.00026	0.0825
0.8	0.0155	0.0194	0.000154	0.0351
2	0.0146	0.0033	0.000121	0.0168
